# miR-192 suppresses leptomeningeal dissemination of medulloblastoma by modulating cell proliferation and anchoring through the regulation of *DHFR*, integrins, and *CD47*

**DOI:** 10.18632/oncotarget.6227

**Published:** 2015-10-25

**Authors:** Seung Yeob Yang, Seung Ah Choi, Ji Yeoun Lee, Ae-Kyung Park, Kyu-Chang Wang, Ji Hoon Phi, Eun Jung Koh, Woong-Yang Park, Sung-Hye Park, Do Won Hwang, Hee Won Jung, Seung-Ki Kim

**Affiliations:** ^1^ Department of Neurosurgery, Dongguk University Ilsan Hospital, Dongguk University, Seoul, Korea; ^2^ Division of Pediatric Neurosurgery, Pediatric Clinical Neuroscience Center, Seoul National University Children's Hospital, Seoul, Korea; ^3^ Department of Neurosurgery, Seoul National University Hospital, Seoul National University College of Medicine, Seoul, Korea; ^4^ Department of Anatomy, Seoul National University College of Medicine, Seoul, Korea; ^5^ College of Pharmacy, Sunchon National University, Jeonnam, Korea; ^6^ Department of Molecular Cell Biology, Sungkyunkwan University School of Medicine, Suwon, Korea; ^7^ Translational Genomics Laboratory, Samsung Genome Institute, Samsung Medical Center, Seoul, Korea; ^8^ Department of Pathology, Seoul National University College of Medicine, Seoul, Korea; ^9^ Department of Nuclear Medicine, Seoul National University College of Medicine, Seoul, Korea

**Keywords:** medulloblastoma, microRNA-192, integrins, CD47, dihydrofolate reductase

## Abstract

**Background:**

The main cause of death in medulloblastoma is recurrence associated with leptomeningeal dissemination. During this process, the role of microRNAs (miRs) in the acquisition of metastatic phenotype remains poorly understood. This study aimed to identify the miR involved in leptomeningeal dissemination and to elucidate its biological functional mechanisms.

**Materials and methods:**

We analyzed the miR expression profiles of 29 medulloblastomas according to the presence of cerebrospinal fluid (CSF) seeding. Differentially expressed miRs (DEmiRs) were validated in 29 medulloblastoma tissues and three medulloblastoma cell lines. The biological functions of the selected miRs were evaluated using *in vitro* and *in vivo* studies.

**Results:**

A total of 12 DEmiRs were identified in medulloblastoma with seeding, including miR-192. The reduced expression of miR-192 was confirmed in the tumor seeding group and in the medulloblastoma cells. Overexpression of miR-192 inhibited cellular proliferation by binding *DHFR*. miR-192 decreased cellular anchoring via the repression of *ITGAV, ITGB1, ITGB3*, and *CD47*. Animals in the miR-192-treated group demonstrated a reduction of spinal seeding (*P* < 0.05) and a significant survival benefit (*P* < 0.05).

**Conclusions:**

Medulloblastoma with seeding showed specific DEmiRs compared with those without. miR-192 suppresses leptomeningeal dissemination of medulloblastoma by modulating cell proliferation and anchoring ability.

## INTRODUCTION

Medulloblastoma is one of the most common malignant brain tumors and a leading cause of cancer-related morbidity and mortality in children. Leptomeningeal dissemination, a potent marker for poor prognosis, is found in up to 40% of children at diagnosis and in most children at recurrence [1]. The tight correlation between leptomeningeal dissemination and poor prognosis for medulloblastoma patients heightens the need to understand the genetic determinants of leptomeningeal dissemination. Substantial progress has been made in recent years in the molecular understanding of medulloblastoma. Four major subgroups can be currently distinguished: WNT, SHH, group 3, and group 4 [2]. Moreover, these molecular subgroupings are related to distinct patient demographics, histologic subtypes, genetic variations, and prognosis. For example, patients with group 3 tumors tend to be younger or male, have anaplastic histology, and are associated with a higher incidence of metastasis [2, 3].

microRNAs (miRs) are a naturally occurring class of small non-coding regulatory RNA that modulate protein expression by binding to the 3′-untranslated region (3′-UTR) of mRNA, inhibiting mRNA translation and affecting transcription [4]. Deregulation of miRs was discovered to play an important role in regulating the expression of various oncogenes and tumor suppressors in a wide variety of human cancers; oncogenic miRs are up-regulated while tumor suppressor miRs are down-regulated in cancer [5]. In recent studies, miR-21 suppression was shown to impede medulloblastoma cell migration, whereas miR-182 promoted leptomeningeal dissemination of non-SHH-medulloblastoma [6, 7]. miR-199b-5p is described as up-regulated in non-metastatic medulloblastomas, and its high expression is associated with better overall survival [8]. However, the molecular mechanisms of miR-mediated medulloblastoma metastasis are still largely unknown. To identify the specific roles of miRs, we investigated the contribution of miRs to tumor seeding using miR microarray profiling in two antithetic groups: one medulloblastoma group with tumor seeding group and one medulloblastoma group without seeding. We then performed *in vitro* and *in vivo* studies to assess the mechanisms of the selected miR in cerebrospinal fluid (CSF) seeding.

## RESULTS

### miR-192 is down-regulated in the tumor seeding group and in medulloblastoma cells

**Figure 1:**
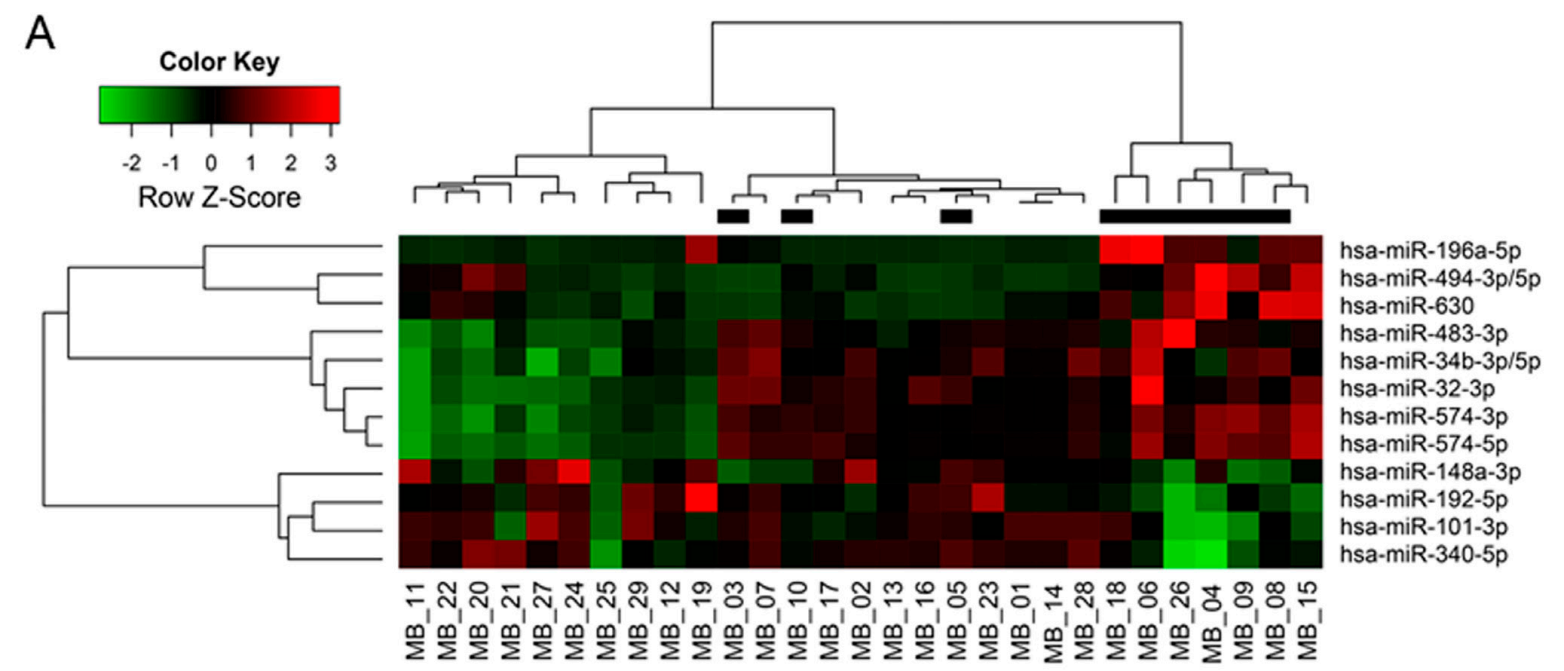

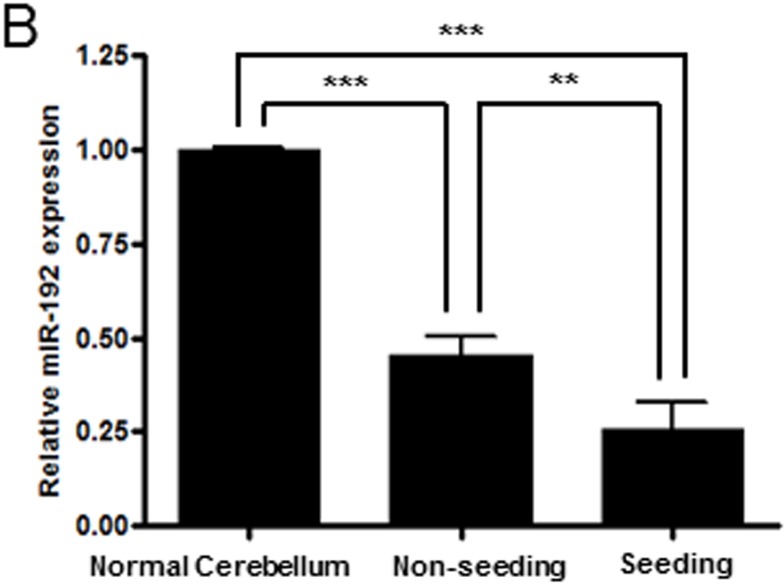

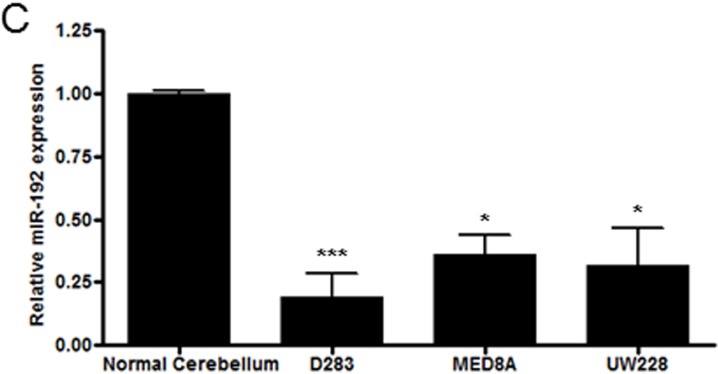
DEmiRs and miR-192 expression **A.** Heatmap of 12 differentially expressed miRs between the seeding and non-seeding medulloblastoma groups. Black bars at the top of the heatmap indicate the presence of seeding. **B.** The expression level of miR-192 is found to be significantly lower in the tumor seeding group (*N* = 9) compared to the tumor non-seeding group (*N* = 20) or to normal cerebellum. **C.** miR-192 expression in medulloblastoma cells. *P < 0.05; **P < 0.01; ***P < 0.001. Error bars represent ± SD (standard deviation).

From analyzing miR expression data between the tumor seeding group and the tumor non-seeding group, we found 12 DEmiRs with minimum log2 expression greater than 5 and range of expression greater than 2 (all *P* values <0.05, Supplementary Table S1 and Figure 1A). Of these DEmiRs, miR-101, -148a, -192, and -340 were significantly lower in expression in the tumor seeding group than in the tumor non-seeding group. Out of the 4 under-expressed DEmiRs, miR-101, -148a, and -340 were over-expressed in medulloblastoma tissues compared to normal cerebellum and/or cortical dysplasia. Similar to a previous study [9], miR-192 was under-expressed in the medulloblastoma tissues compared to the normal cerebellum and the cortical dysplasia (Supplementary Figure 1). Therefore, we focused on biological function of miR-192. We found that the expression level of miR-192 was significantly lower in the tumor seeding group (*N* = 9) compared to the tumor non-seeding group (*N* = 20) or the normal cerebellum group (all *P* values <0.05, Figure 1B). We confirmed the lower expression level of miR-192 in all medulloblastoma cells compared to the normal cerebellum using real-time qRT-PCR (all *P* values <0.05, Figure 1C).

### Transfection of miR-192 in medulloblastoma cells

To determine the functional significance of miR-192, all medulloblastoma cells were transfected with miR-192. After transfection, miR-192 levels significantly increased compared to negative control (NC) miR, respectively (all *P* values <0.05, Supplementary Figure 2A and 2B).

### Overexpression of miR-192 suppresses cellular viability and proliferation and increases cell cycle arrest

**Figure 2:**
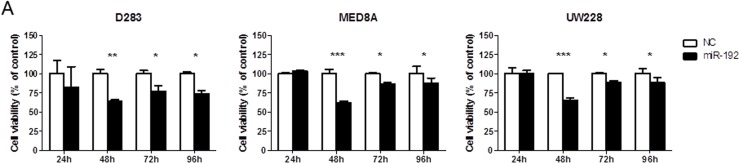

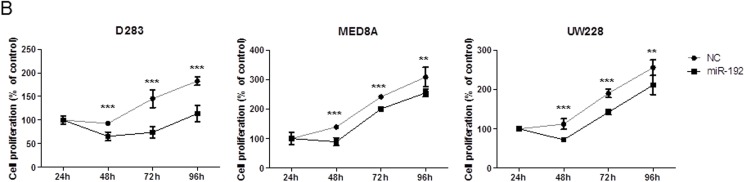

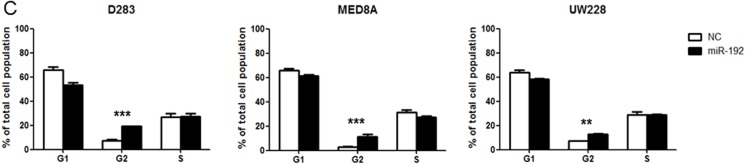
miR-192 and cell proliferation **A.** cell viability assay for 96 hours. **B.** BrdU cell proliferation assay for 96 hours. Overexpression of miRNA-192 decreased cellular proliferation. **C.** Cell cycle assay. Overexpression of miR-192 induced cell cycle G2 arrest. *P < 0.05; **P < 0.01; ***P < 0.001. Error bars represent ± SD.

Overexpression of miR-192 suppressed cellular viability (all *P* values <0.05, Figure 2A) and proliferation (all *P* values <0.01, Figure 2B) at 48, 72, and 96 hours in medulloblastoma cells. The NC miR had no effect on cellular viability or proliferation, suggesting that miR-192-mediated inhibition of cellular viability and proliferation is specific to miR-192. Cell cycle analysis using flow cytometry revealed a significant increase in the fraction in G2 phase after miR-192 transfection compared with controls (all *P* values <0.01, Figure 2C).

### *DHFR* is a downstream target of miR-192

**Figure 3:**
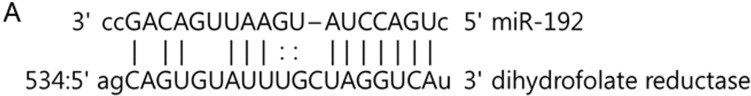

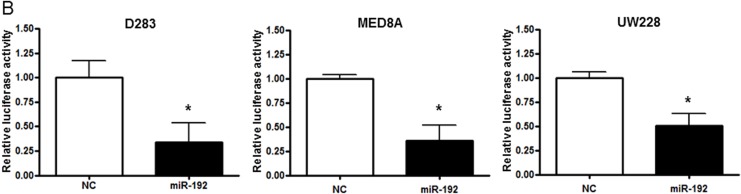

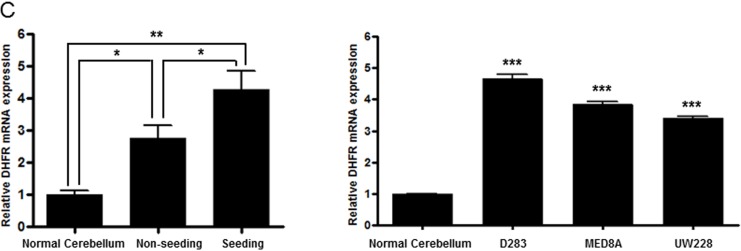

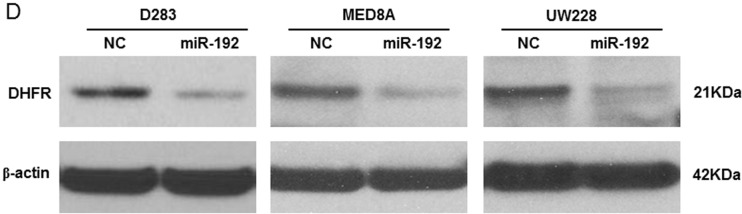

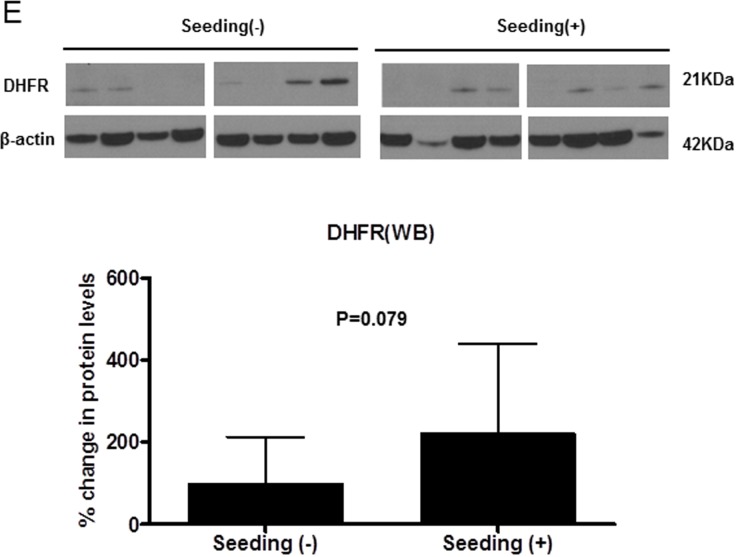

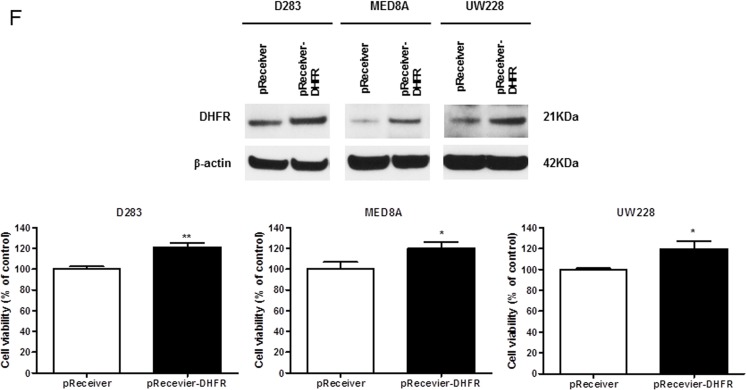
miR-192 binds dihydrofolate reductase (DHFR) **A.** miR-192 binding site at the 3′-UTR of *DHFR* mRNA. **B.** Analyzing miR-192 targets with a luciferase reporter shows that *DHFR* is a target of miR-192. **C.** The expression of *DHFR* mRNA in normal cerebellum, tumor tissues, and medulloblastoma cells. **D.** Western blot analysis of the expression of DHFR protein in medulloblastoma cells transfected with miR-192. DHFR protein expression is significantly decreased by miR-192 transfection in all medulloblastoma cells. **E.** Expression of DHFR in medulloblastoma tissues (*N* = 16). **F.** Western blot analysis shows the overexpression of DHFR. Overexpression of DHFR increases the cellular viability (pReceiver vector *vs*. pReceiver-DHFR: 100.0 ± 4.7 % *vs*. 120.1 ± 6.4 %, P = 0.001 in D283; 100.0 ± 11.7 % *vs*. 117.4 ± 13.1 %, *P* = 0.004 in MED8A; 100.0 ± 2.5 % *vs*. 119.6 ± 16.5 %, *P* = 0.043 in UW228). *P < 0.05; **P < 0.01; ***P < 0.001. Error bars represent ± SD.

DHFR is a target of methotrexate and the key enzyme responsible for intracellular folate metabolism, which is essential for DNA and RNA synthesis [10]. Based on structural analysis of *DHFR's* 3′-UTR miR target analysis (http://www.targetscan.org/ and http://www.microrna.org/) and a previous study [11], we identified miR-192 as potentially interacting with the 3′-UTR region of *DHFR* mRNA (Figure 3A).

Then, we cloned the *DHFR* 3′-UTR fragment containing this predicted site into the pSiCHECK2 luciferase reporter. Co-transfection of pre-miR-192 and pSiCHECK2-*DHFR* significantly decreased the luciferase activity compared with the control in all medulloblastoma cells (all *P* values <0.05, Figure 3B), indicating that *DHFR* is a target of miRNA-192.

We explored the mRNA and protein levels of DHFR in medulloblastoma tissues and in three medulloblastoma cell lines. *DHFR* mRNA was higher in the seeding group than in the normal cerebellum and the non-seeding group (all *P* values <0.05, Figure 3C). *DHFR* mRNA level was higher in all of the medulloblastoma cell lines than in the normal cerebellum (all *P* values <0.05, Figure 3C). Western blot analysis showed that overexpression of miR-192 decreased the expression of DHFR in all of the medulloblastoma cell lines (Figure 3D) and DHFR were higher in the seeding group (*N* = 8) compared with the non-seeding group (*N* = 8) (Figure 3E).

### Overexpression of DHFR increases the cell viability of medulloblastoma cells

To confirm the biological function of the overexpression of DHFR on cellular viability, we transfected pReceiver-DHFR into the medulloblastoma cells. Overexpression of DHFR significantly increased the cell viability (all *P* values <0.05, Figure 3F).

### miR-192 does not influence invasion or EMT in medulloblastoma cells

**Figure 4:**
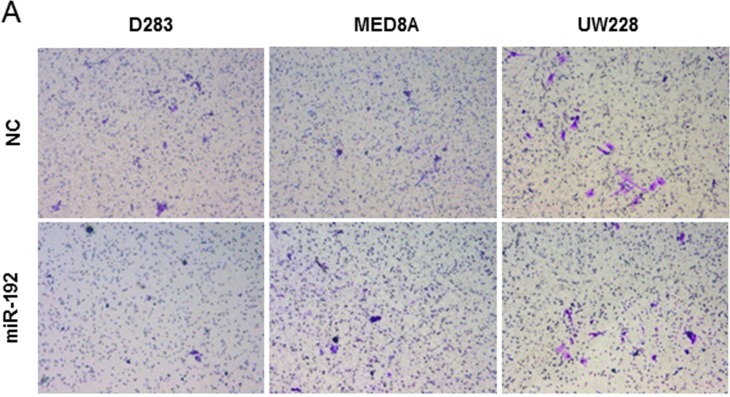

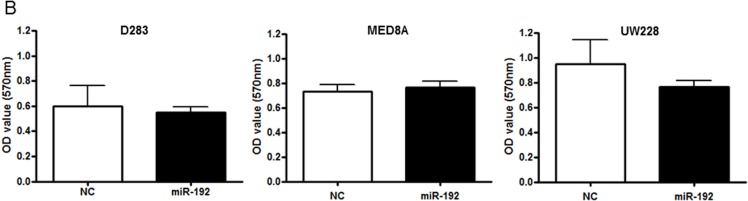

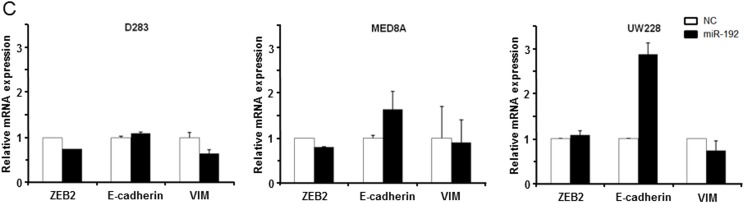

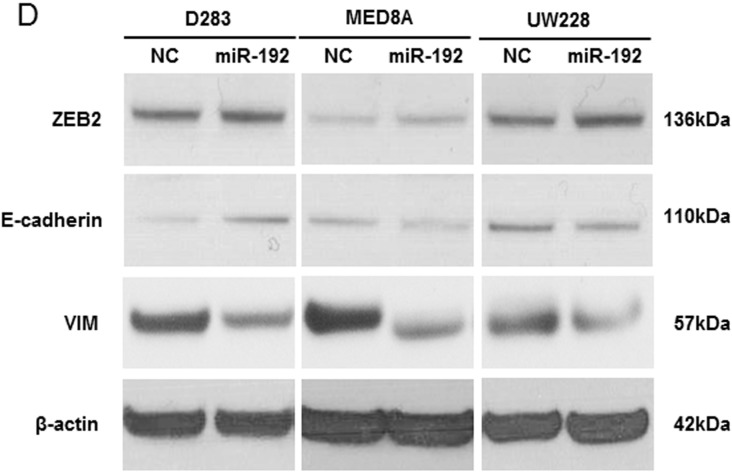
miR-192 and epithelial-mesenchymal transition (EMT) **A.** and **B.** miR-192 does not activate the cell invasion capability of medulloblastoma cells compared with normal control (NC) miR. **C.** and **D.** EMT-related mRNA and protein expression after miR-192 transfection.

The role of miR-192 in medulloblastoma cell invasion was investigated. Following miR-192 or NC-miR transfection in all medulloblastoma cell lines, no significant differences were observed in the percentage of migrated cells (Figure 4A). There was no significant impact on the optical density of migrated medulloblastoma cells from transfection with miR-192 versus NC-miR (all *P* values >0.05, Figure 4B). These results showed that miR-192 did not influence cell invasion behavior.

Next, we transfected the medulloblastoma cells with miR-192 and analyzed EMT-related genes and proteins such as ZEB2, E-cadherin and VIM after miR-192 transfection (Figure 4C). *ZEB2* mRNA and protein expression were not stimulated by the overexpression of miR-192 in any of the medulloblastoma cell lines. The expression of E-cadherin mRNA increased but protein expression did not change in the MED8A and UW228 lines. In D283, E-cadherin mRNA and protein expressions increased slightly. Notably, VIM protein was markedly diminished even though its mRNA expression was only slightly reduced in miR-192 overexpressing medulloblastoma cells (Figure 4D).

### Overexpression of miR-192 inhibits the adhesive capability of medulloblastoma cells

**Figure 5:**
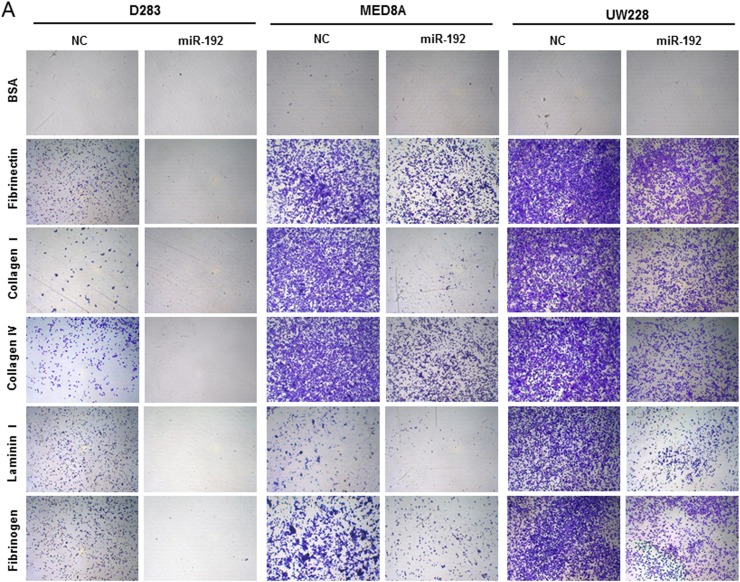

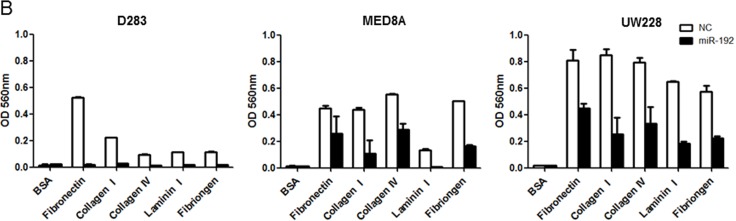
miR-192 and cell anchoring Overexpression of miR-192 inhibits the adhesive capability of medulloblastoma cells. Adherent cells are stained **A.** and quantified at OD 560 nm after extraction **B.**.

To determine the functional role of miR-192 in cell adhesion, we performed an adhesion assay after miR-192 transfection. Overexpression of miR-192 significantly inhibited adhesion to fibronectin, collagen type I, collagen type IV, laminin type I and fibrinogen in all medulloblastoma cells compared to the NC miR (all *P* values <0.05, Figure 5A and 5B). These results suggest that miR-192 inhibits cell attachment to the ECM.

### miR-192 binds the 3′-UTR of *ITGAV, ITGB1, ITGB3,* and *CD47* and represses their expression

**Figure 6:**
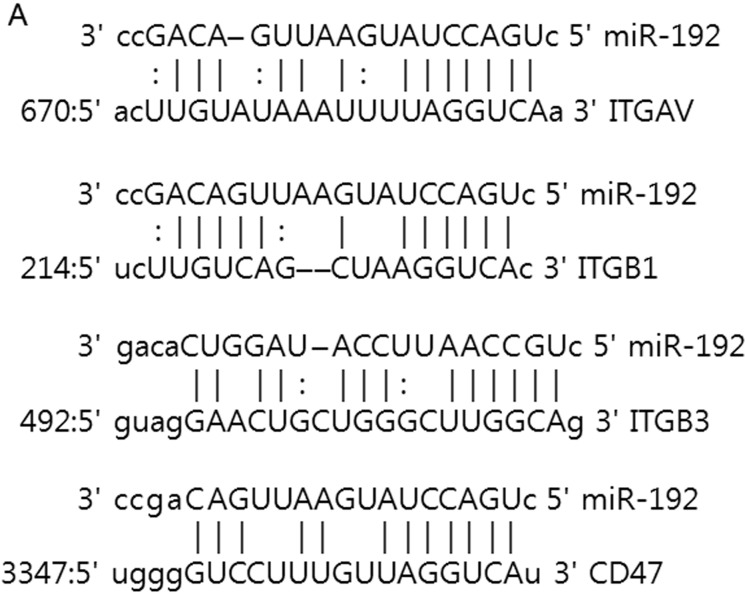

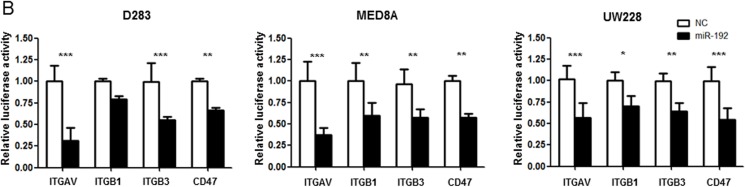

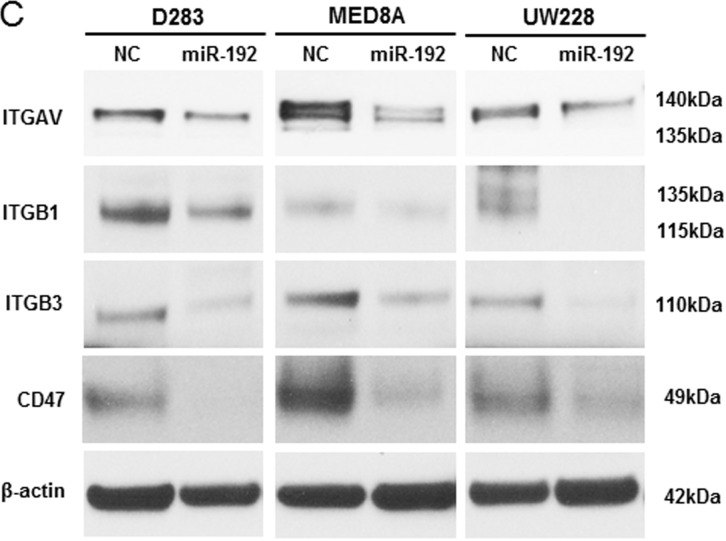

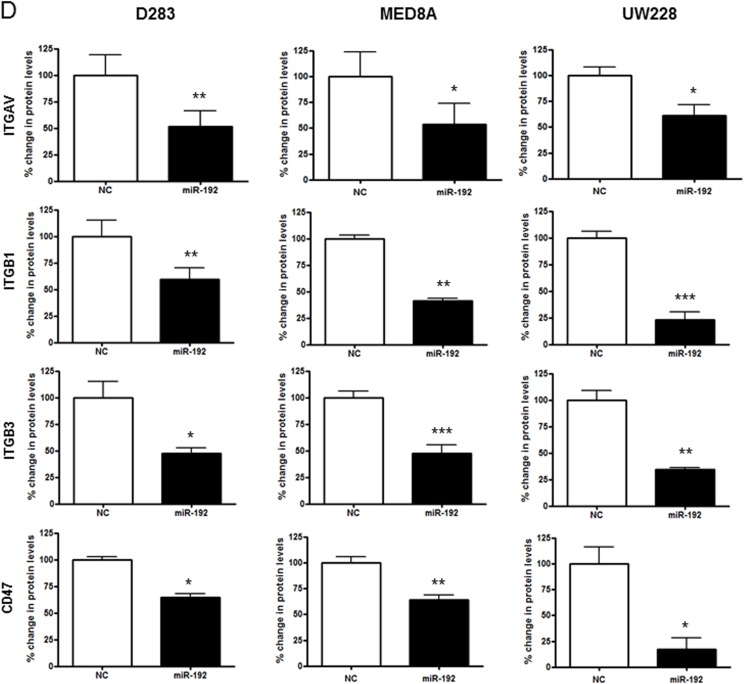

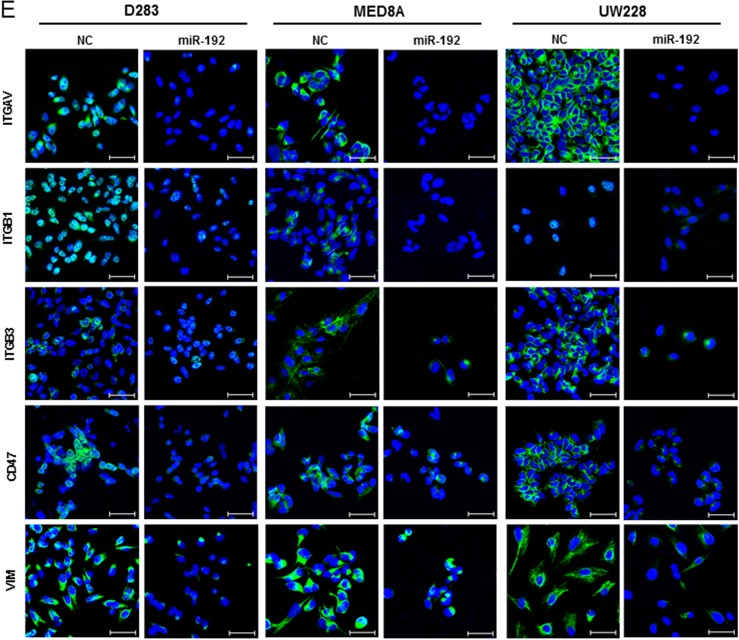

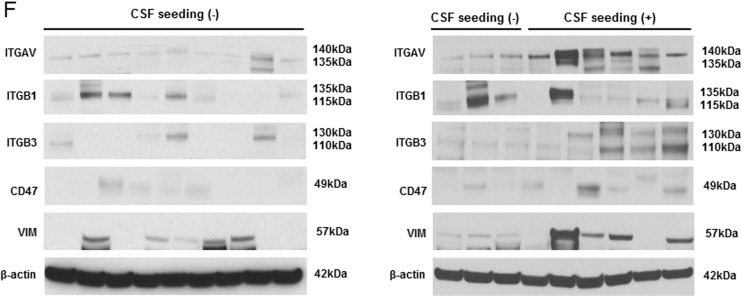

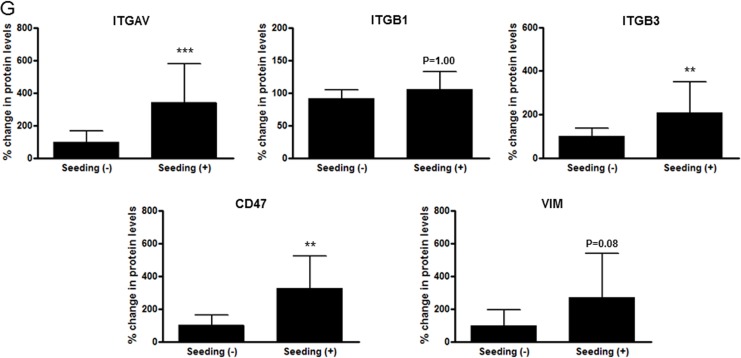
miR-192 binds specific α and β integrin subunits and *CD47* **A.** Alignment of miR-192's seed sequence with candidate binding sequences within the 3′-UTRs of the indicated integrin subunits (αV, β1, β3, and *CD47*). **B.** overexpression of miR-192 results in significant reduction in luciferase activity for *ITGAV*. **C.** and **D.** Western blot analysis shows that miR-192 transfectants show a significant reduction in the expression of *ITGAV*, *ITGB1*, *ITGB3*, and *CD47*. Quantitation of western blot bands: ITGAV, ITGB1, ITGB3, and CD47. **E.** Regulation of integrin subunits and related proteins by miR-192. Representative immunofluorescence staining showing the localization of ITGAV, ITGB1, ITGB3, CD47, and VIM in response to miR-192 transfection. Scale bar indicates 50 μm. *P < 0.05; **P < 0.01; ***P < 0.001. Error bars represent ± SD. miR-192 inhibits the expression of *ITGAV*, *ITGB1*, *ITGB3*, *CD47*, and *VIM* in medulloblastoma. **F.** Western blot analysis of integrin subunits and CD47 expression between the seeding and non-seeding groups. **G.** Quantitation of the western blot bands. ITGAV, ITGB3 and CD47 expression are significantly lower in the seeding group, but ITGB1 and VIM are not different between the seeding and non-seeding groups.

Medulloblastoma disseminates through the CSF in the leptomeningeal space to coat the brain and spinal cord [12, 13]. CSF flow serves as a ‘transporter’ for tumor cells; therefore, cell motility itself may not be important in CSF seeding, unlike glioma invasion. We speculated that acquisition of an enhanced adhesion capacity for the binding site and proliferation of tumor cells could be more important than migratory and invasive capacity for the dissemination of medulloblastoma. As integrins and integrin-related proteins are central regulators of focal adhesion dynamics, changes in their expression resulting from miR deregulation in tumors represent a functionally relevant contribution to metastatic dissemination [14, 15]. By *in silico* analyses (http://www.targetscan.org/ and http://www.microrna.org/), we identified 4 genes (*ITGAV, ITGB1, ITGB3,* and *CD47*) that are potentially regulated by miR-192 (Figure 6A). We then conducted luciferase reporter gene assays to show that the posttranscriptional repression of these 4 genes is a consequence of miR-192 binding to binding seed sequences within the 3′-UTRs of their transcripts. In cells transfected with these 4 genes' 3′-UTR-containing vectors, overexpression of miR-192 resulted in a significant reduction in luciferase activity for all (all *P* values <0.05, Figure 6B) except ITGB1 in D283 (*P* value = 0.08).

To confirm the regulation of these 4 genes by miR-192, we transfected the medulloblastoma cells with miR-192. After miR-192 transfection, there was a significant reduction in ITGAV, ITGB1, ITGB3, and CD47 in all miR-192-transfected medulloblastoma cells in comparison with NC miR-transfected cells (all *P* values <0.05, Figures 6C and 6D). Furthermore, miR-192 decreased the protein expression of ITGAV, ITGB1, ITGB3, and CD47 and induced nuclear accumulation of ITGAV, ITGB1, ITGB3, and CD47 (Figure 6E).

### miR-192 decreases ITGAV, ITGB1, ITGB3, and CD47 expression in medulloblastoma tissues

To determine whether integrin subunits are related to the dissemination of medulloblastoma, their protein levels were analyzed in the seeding group (*N* = 8) and non-seeding group (*N* = 10). Compared with the seeding group, ITGAV (*P* <0.001), ITGB3 (*P* <0.01) and CD47 (P <0.01) were significantly lower in the non-seeding group. ITGB1 (*P* = 1.00) and VIM (*P* = 0.08) were not (Figure 6F and 6G).

### miR-192 inhibits leptomeningeal seeding in a nude mouse xenograft model

**Figure 7:**
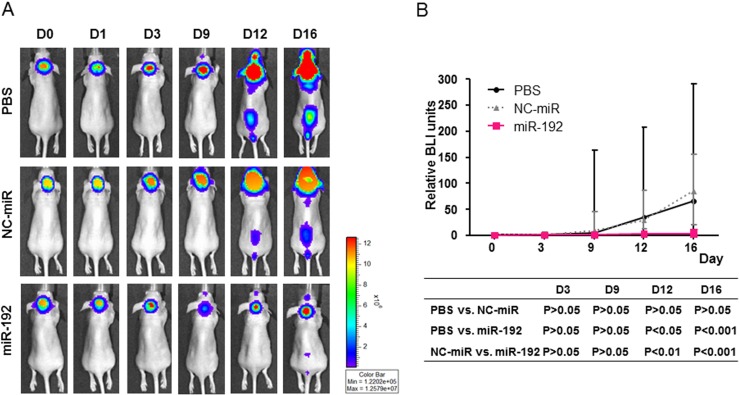

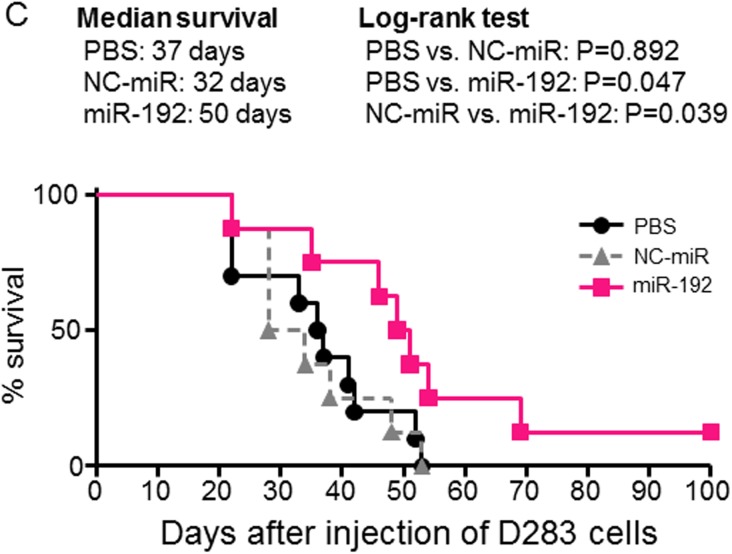
Therapeutic effect of miR-192 and inhibition of tumor seeding *in vivo* **A.** Representative live *in vivo* imaging of implanted D283-effLuc cells treated with PBS, NC-miR, or miR-192 by intranasal injection for 16 days. **B.** Serial measurement of tumor-occupied areas in live in vivo imaging. **C.** Kaplan–Meier survival plots of mice implanted D283-effLuc cells reveals the increased survival by miR-192.

D283-effLuc cells were successfully conducted in all mice without mortality. Live in vivo bioluminescence imaging (BLI) of the mice treated with PBS or with NC-miR revealed a progressive enlargement of primary tumor for 9 days and seeding along the spinal cord thereafter. In contrast, the mice treated with miR-192 exhibited stable primary tumor for 12 days and minimal seeding along the spinal cord thereafter (Figure 7A). A significant difference in the BLI signal areas between the groups (PBS or NC-miR vs. miR-192) was observed (Figure 7B).

Furthermore, we confirmed the therapeutic efficacy of miR-192 by survival analysis. A Kaplan-Meier survival curve demonstrated a significant survival benefit in the mice treated with miR-192 (median survival, 50 days) compared to PBS (median survival, 37 days; *P* = 0.047) or NC-miR (median survival, 32 days; *P* = 0.039) treated groups (Figure 7C). There was no statistically significant difference in the survival between the mice treated with PBS and the mice with NC-miR (*P* = 0.892) (Figure 7C).

## DISCUSSION

We identified 12 DEmiRs between the tumor seeding group and tumor non-seeding group in medulloblastoma. Among these, we focused on miR-192 because it was down-regulated in medulloblastoma tumor samples compared to normal cerebellum and has been known to regulate EMT [9, 16, 17]. Although miR-192 does not decrease cell invasion directly, we found that miR-192 inhibits *DHFR*-related cell proliferation and integrin-related cell anchoring. This is the first report outlining the phenocopying effects of miR-192 down-regulation on leptomeningeal dissemination of medulloblastoma *in vitro* and *in vivo*.

Morfouace *et al*. performed high-throughput screening of a bioactive library consisting of 7,389 compounds including 830 US Food and Drug Administration-approved drugs, and they identified gemcitabine and pemetrexed as efficacious at increasing the survival of mice bearing patient-derived xenograft Group 3 medulloblastomas in which MYC is overexpressed [18]. Pemetrexed inhibits DHFR in the folate pathway, which is essential for the rapid cellular division and proliferation of cancer cells [19]. Hence, the inhibition of *DHFR* can limit the growth and proliferation of cells, and the expression of DHFR can be regulated, at least in part, at the translational level [11, 20]. A previous study showed that miR-24 has a binding site in the 3′-UTR of *DHFR* mRNA and that a single nucleotide polymorphism in miR-24 results in the loss of miR-24-mediated inhibition of *DHFR*, in methotrexate resistance, and is associated with an increase in *DHFR* mRNA and protein [20]. Other studies have shown that miR-192 inhibits *DHFR* and cell proliferation through the p53-miR circuit and is down-regulated in metastatic cancers [11, 21]. However, the role of miR-192 and the contribution of *DHFR* in medulloblastoma have not been fully explored, and most of their overall biological functions remain unknown. We found that miR-192 significantly suppresses *DHFR* expression and that DHFR expression is higher in the tumor seeding group than in the non-seeding group. Like miR-24, in the present study, miR-192 overexpression suppresses cell viability and cell proliferation and restores cell cycle control by suppressing *DHFR* expression [11]. These results provide further evidence that miR-192 is one of the candidate miRs involved in the suppression of a key cancer gene, *DHFR*.

In various cancers, cancer cells can undergo EMT to escape from the primary tumor, invade surrounding tissues, and eventually colonize remote sites to generate metastases [22]. Initially, we hypothesized that metastatic dissemination of medulloblastoma might be associated with EMT. Although VIM was reduced by miR-192 transfection in all medulloblastoma cells, EMT-related proteins such as ZEB2 and E-cadherin were not affected. miR-192 also did not inhibit the cell invasion of medulloblastoma cells. In contrast, miR-192 suppressed cell adhesion by binding *ITGAV* and *ITGB3*. *ITGB3* is mostly associated with the ability of tumor metastases to promote extravasation from the primary tumor, cell adhesion, intravasation, and tumor growth at the metastatic site [23, 24]. VIM interacts with ITGB3 and plectin, which together regulate the organization and distribution of VIM in several different cell types. A possible role for ITGB3-mediated recruitment of VIM to the cell surface is to increase the adhesive strength of the cells binding to the substrate [25]. The cells' adhesion ability positively correlates with ITGAVB3, indicating an increase in their metastatic potential [26, 27]. Based on our findings and those of others [23-27], the role of miR-192 in metastatic dissemination of medulloblastoma may be more related to cell adhesion than to EMT at the metastatic site.

Metastatic cells have to induce angiogenesis to escape the limitations of passive diffusion of nutrients and oxygen, which hamper metastatic colonization and growth [28]. ITGB3 plays an important role in tumor-induced angiogenesis and has been described as a pro-angiogenic factor [23, 26, 29, 30]. Therefore, miR-192-mediated ITGB3 (especially ITGAVB3) plays an important role in tumor-induced angiogenesis, which is essential for metastatic colonization and growth.

Although there was no significant difference in ITGB1 levels between the tumor seeding group and the tumor non-seeding group in the present study, miR-192 contributed to decreased ITGB1 expression in all miR-192-transfected medulloblastoma cells. ITGB1, known as a part of the focal adhesion platform, helps to stabilize cell attachment to ECM ligands [31]. Binding of ITGB1 to collagen results in downstream rearrangements of the actin cytoskeleton by providing a scaffold for cytoskeletal proteins and multiple signaling molecules that are involved in the regulation of cell adhesion and spreading [32]. Our findings showed that miR-192-mediated inhibition of *ITGB1* expression results in decreased cell anchoring ability during the leptomeningeal dissemination of medulloblastoma.

CD47, which has been shown to interact with and stimulate ITGAVB3 activation, co-localizes with E-cadherin at cell-cell adhesion sites and participates in the regulation of cell-cell adhesion and cell migration through reorganization of the actin cytoskeleton in epithelial cells [29, 33, 34]. Aberrant expression of miRs has been implicated in deregulating the expression and activity of integrin and *CD47*, leading to the development and progression of primary tumors, including their acquisition of the metastatic phenotype. Blockade of CD47 by neutralizing antibodies reduces migration and chemotaxis in response to some cancer-derived cells [33, 34]. Our results indicated that miR-192-mediated CD47 suppression can inhibit leptomeningeal dissemination of medulloblastoma through molecular cross talk with ITGAVB3. Recent studies have also demonstrated that the CD47-signal regulatory protein α (SIRPα) signaling system plays important roles in tumor immune surveillance through regulation of the phagocytic activity of macrophages. Blocking this signaling system enhances macrophage-mediated clearance of tumor cells [35, 36]. Although we did not determine whether miR-192 regulates the CD47-SIRPα immune surveillance system, this will be an area for future research.

Together with the results of other studies [21, 37], our results suggest that miR-192 is a metastasis suppressor gene whose pleiotropic functions inhibit multiple steps of the cancer metastasis cascade by binding prometastatic genes including integrins. This pleiotropic effect of miR-192 is of potential interest from a translational perspective because modulation of a single miRNA, for example via adeno-associated viral vectors or synthetic miRNA precursors [38], can inhibit the function of several genes involved in cancer metastasis.

Our study has some limitations that need to be considered. First, all of the medulloblastoma tissues in the seeding group were taken from the primary tumor site. Therefore, the medulloblastoma tissues in the seeding group may or may not be the same as tumors in seeding area. The desirable method for evaluating the effect of miR-192 on tumor seeding would be assessing tumors in two groups: patients' metastatic tissues in seeding group and primary tumors in non-seeding group. However, it is not clinically considered to get metastatic tissues from seeding area. In the light of improved adjuvant treatment modalities such as chemotherapy or radiotherapy, metastatic tissues in the seeding area do not have to be resected to prevent sequela. Glioma disseminates mainly through white matter tracts whereas medulloblastoma usually disseminates through the subarachnoid space via the CSF flow. Increased ability of cell anchoring according to the loss of miR-192 expression may make it more difficult to detach tumor cells from primary site to CSF. However, medulloblastomas are in direct contact with the CSF and it is possible to detach the tumor tissues by chance. Therefore, acquisition of an enhanced adhesion capacity for the binding site and then proliferation of tumor cells could be more important than migratory and invasive capacity for the dissemination of medulloblastoma. Nonetheless, *in vitro* and *in vivo* studies provide valuable information that miR-192 suppresses leptomeningeal dissemination of tumor by regulating cellular proliferation and cell anchoring. Second, in the present study, medulloblastoma cells are transiently transfected but there are pros and cons in this protocol. The main disadvantage of transient transfection is that the nucleic acids are expressed for a short period of time. Clinically, there is a limitation in adopting stable transfection of tumor cells with miR. To overcome these obstacles, we repeated intranasal injections of miR-192 and confirmed their effect through *in vivo* study. Therefore, repeated intranasal injections of miR-192 can be a practically useful method.

In summary, this study revealed that the loss of miR-192 expression exerts wide ranging effects on cell proliferation and cell anchoring by activating *DHFR* and by molecular cross talk between *ITGAV, ITGB1, ITGB3,* and *CD47* in the leptomeningeal dissemination of medulloblastoma.

## MATERIALS AND METHODS

### Tissue samples

Twenty-nine frozen medulloblastoma tissues were collected from the Brain Bank of the Division of Pediatric Neurosurgery, Seoul National University Children's Hospital (SNUCH). Patient selection was based on the availability of snap-frozen tissues sufficiently abundant for this study. The individual that selected the patients was blind to their clinical information except the diagnosis. No previous irradiation or systemic chemotherapy had been conducted on these patients prior to microsurgery. Detailed information is listed in Supplementary Table S2. To assess the molecular biological processes for tumor dissemination, the 29 medulloblastoma tissues were divided into two groups: a tumor seeding group (*N* = 9) and a tumor non-seeding group (*N* = 20). We defined tumor seeding according to our previous study [39]. All of the samples were gathered according to the Institutional Review Board–approved protocol and with written informed consent from each child's parents or guardians.

### miR microarray profiling and data analysis

Total RNA isolation and small RNA (including miR) enrichment were performed on medulloblastoma tissues using the mirVana^TM^ miRNA Isolation Kit (Ambion). The Agilent Human miRNA Microarray Kit (V2) containing probes for 723 human miRs was used for miR microarray chip hybridization. RNA labeling and hybridization were performed according to the manufacturer's protocol. Microarray images were scanned with the Agilent microarray scanner. Total gene signals were extracted using Agilent Feature Extraction software. A small constant, 16, was added to the scanned raw expression values to ensure that all expression values were greater than zero before log_2_ transformation. The log_2_-transformed data were normalized by the quantile normalization method using R/Bioconductor [40]. To identify differentially expressed miRs (DEmiRs) between the tumor seeding group and the tumor non-seeding group, Bayesian moderated t-statistics were computed [41]. miR-expression datasets can be accessed at www.ncbi.nlm.nih.gov/geo (accession no. GSE66968).

### Cell culture

Human medulloblastoma cell line D283 was purchased from ATCC, and MED8A and UW228 were provided by Dr. Young Shin Ra (Asan Medical Center, Seoul, Korea). D283 cells were cultured in Eagle's minimum essential medium (ATCC), and MED8A and UW228 cells were cultured in Dulbecco's modified Eagle's medium (Welgene). All media were supplemented with 10% FBS (Invitrogen) and penicillin-streptomycin (Invitrogen). All medulloblastoma cells were grown at 37°C in a humidified atmosphere of 5% CO_2_.

### Transfection of miRs in medulloblastoma cells

Precursor miR-192 and negative control (NC) miR (Ambion) (50nM) were transfected into the three medulloblastoma cell lines using Lipofectamine RNAiMAX (Invitrogen) according to the manufacturer's instructions. The transfected cells were harvested at the indicated days and used for further studies. Cy3-labeled NC miR (Ambion) was used to observe transfection efficacy by fluorescence microscope.

### Real-Time qRT-PCR analysis of mRNA and miR

Total RNA, including miRs, was isolated from tissues and cells as described previously. Normal human cerebellum total RNA was purchased from Clontech Laboratories.

miR and mRNA expression were confirmed with TaqMan probes (Applied Biosystems) using the Applied Biosystems 7500 Real-time PCR system according to the manufacturer's protocol. The reactions were performed under conditions specified in the ABI TaqMan Gene Quantitation assay protocol and were repeated in triplicate. Signals were collected at the endpoint of every cycle. The gene expression delta cycle threshold values of miRs and mRNA from each sample were calculated by normalizing to an internal control, RNU6B or GAPDH, and relative quantitation values were plotted.

### Cell viability, proliferation and cell cycle assay

Cell viability was assessed with cell counting kit-8 (Dojindo) and proliferation was determined using a BrdU cell proliferation kit (Roche) according to the manufacturer's protocol. Percentage of cell viability was determined by the relative absorbance of the cells transfected with miR-192 versus that of cells transfected with NC miR.

For cell cycle analysis, a standard flow cytometry protocol was performed using propidium iodine (Sigma-Aldrich) as described previously [42]. All experiments were performed in triplicate and repeated at least three times.

### Invasion assay

Invasion was assessed using the QCM ECMatrix Cell Invasion Assay (24-well, 8μm, colorimetric kit, Millipore) according to the manufacturer's protocol. After transfection, cells (5 × 10^4^/well) in 300μl serum-free medium were added to the upper chamber. Then, 500μl of 10% FBS-containing medium was added to the lower chamber as a chemoattractant. All experiments were performed in triplicate.

### Adhesion assay

Cell adhesion was assessed using the CytoSelect 48-well cell adhesion assay kit (Cell Biolabs) according to the manufacturer's protocol. After transfection, cells (1 × 10^5^/well) in 300μl serum-free medium were seeded in the 48-well plates coated with extracellular matrix (ECM) including fibronectin, collagen type I, collagen type IV, laminin type I, fibrinogen, and bovine serum albumin (BSA). All experiments were performed in triplicate.

### Determination of miR-192 targeted sequences by computational prediction

We performed database searches in miR target prediction engines (http://www.targetscan.org/ and http://www.microrna.org/) to predict the miR-192 binding sequences. Targets predicted by both databases and related to tumor metastasis were considered relevant to our research.

### Dual luciferase miR target reporter assay

The 3′-UTR of dihydrofolate reductase (DHFR) was amplified by PCR using the following primers: 5′-CCG CTCGAGCTTGACATTGTCGGGCTTTT-3′ (forward) and 5′-ATAAGAAT GCGGCCGC TGCAAACACCTGAGACTTGCt-3′ (reverse). The PCR product was extracted from the gel and subsequently cloned into the pSiCHECK-2 Vector (Promega). After sequencing, the construct was verified by sequencing. The 3′-UTR of *ITGAV, ITGB1, ITGB3,* and *CD47* cloned into the pEZX vector and control vectors were purchased from GeneCopoeia. Cells were co-transfected with the 50ng pSiCHECK-2 or pSiCHECK-2-*DHFR* 3′-UTR, 100ng pEZX or pEZX-3′-UTR of *ITGAV, ITGB1, ITGB3*, or *CD47* with 50nM miR-192 or NC miR. Luciferase activity was measured by using a Dual-Glo luciferase assay system (Promega) for the pSiCHECK vector and a Dual-Luciferase Reporter Assay System (Genecopoeia) for the pEZX vector. Relative luciferase activity was calculated by normalizing the firefly luminescence to the Renilla luminescence following the manufacturer's protocol. All experiments were performed in triplicate.

### DHFR overexpression

pReceiver vector control and pReceiver-DHFR were purchased (GeneCopoeia). To introduce pReceiver vector control and pReceiver-DHFR into D283, MED8A, and UW228, electroporation was performed using a Neon Transfection System (Life Technologies) according to manufacturer's instructions. Transfection efficiency was determined 48-hour after transfection by western blot analysis.

### Immunoblotting

Total protein was extracted from tissues and transfected cells using RIPA buffer including protease inhibitor cocktail (Cell Signaling Technology), and western blotting was carried out as described previously [42]. The primary antibodies used were as follows: anti-DHFR (1:400, Cell Signaling Technology), anti-ZEB2 (1:1000, Abcam), anti-E-cadherin (1:250, Abcam), anti-VIM (1:1500, Cell Signaling Technology), anti-ITGAV (1:1000, Cell Signaling Technology), anti-ITGB1 (1:2000, Cell Signaling Technology), anti-ITGB3 (1:2000, Cell Signaling Technology), anti-CD47 (1:2000, Abcam), and anti-β-actin (1:5000, Sigma-Aldrich).

### Immunofluorescence staining

Cells (1 × 10^4^/well) were seeded on 8-well chamber slides (Lab-Tech) after transfection. The cells were fixed, permeated and blocked. Then, they were incubated with the following antibodies: anti-VIM (1:200, Abcam), anti-ITGAV (1:1000, Cell Signaling Technology), anti-ITGB1 (1:1000, Cell Signaling Technology), anti-ITGB3 (1:500, Cell Signaling Technology) or anti-CD47 (1:200, Abcam). The secondary antibody, Alexa Fluor 488-conjugated goat anti-mouse IgG or anti-rabbit IgG (1:500, Invitrogen), was applied for 1 hour. Slides were mounted with antifading solution containing 4′-6′-diamidino-2-phenyl-indole (Vector Laboratories). Images were taken using a confocal microscope (Zeiss). All experiments were conducted in triplicate.

### Retroviral infection for *in vivo* study

To visualize the transplanted D283 cells, D283 cells were transfected with an *effLuc* viral vector and a DNA vector carrying major structural proteins (GAG, Pol, and Env) using lipofectamine 2000 (Invitrogen) as described previously [43]. The infected cells were sorted by magnetic-activated cell sorting (Miltenyi Biotech Ltd.) through monoclonal anti-CD90.1 conjugated to magnetic microbeads.

### *In vivo* leptomeningeal seeding assay in a nude mouse xenograft model

For leptomeningeal seeding of medulloblastoma mouse model, female BALB/c nude mice (7–8 weeks old) (Orient Bio Inc.) were kept under specific pathogen-free conditions. The Institutional Animal Care and Use Committee of Seoul National University Hospital (SNUH) approved all animal experiment protocols (SNUH-IACUC No.5-0156-S1AO). The mice (*N* = 24) were anesthetized by an intraperitoneal injection of 20 mg/kg Zoletil (Virbac) and 10mg/kg Rompun (Bayer Korea). The mouse heads were fixed in a stereotactic guiding device (David Kopf Instruments), and the cisterna magna was exposed under a microscopic view. D283-effLuc (1.2 × 10^6^ cells) cells were slowly injected into the subarachnoid space of the cisterna magna using a 30-guage needle as previously described [44].

Three days after D283-effLuc cells implantation, the mice were randomized into three groups (*N* = 8/group). The mice treated with intranasal injection of disulfide (–S–S–) linkage in the branched PEI (SSPEI)+PBS (PBS), SSPEI+NC-miR (NC-miR), or SSPEI+miR-192 (miR-192) every three days for two weeks. NC-miR or miR-192 was encapsulated with before treatment [45]. The protocol of intranasal delivery was adopted from previously published studies [46, 47].

For the acquisition of the live bioluminescence images, the mice were sedated with 2% isoflurane in 100% O_2_ through a nose cone. D-Luciferin (150 mg/kg, Caliper Life Sciences) was administered into the peritoneal cavity following the manufacturer's protocol. Bioluminescence images were taken by an *In-Vivo* Imaging System 100 (Xenogen Corp.) on 0, 1, 3, 9, 12, and 16 days after cell transplantation. Images were acquired by integrating light for 1–3 minutes and the luminescence intensity in regions of interest (ROI) from each image was quantified to examine the viability of the implanted cells. The signal was quantified in units of photons per second per square centimeter per steradian (photons/s/cm^2^/sr). Results were presented as fold change over bioluminescence measured at the start of treatment [48]. The mice were monitored daily for neurologic symptoms until they were euthanized.

For overall survival analysis, survivals were followed until the mice were dead or for a maximum of 100 days. The development of symptoms requiring euthanasia was considered as mortality and all euthanized rats were verified as bearing tumors by necropsy.

### Statistical analysis

Values are presented as the mean ± standard deviation (SD). Statistical significance was evaluated by Student's t-test and χ2-test for comparisons between two groups of data. *P* < 0.05 was accepted as significant and indicated a significant difference in the experimental groups compared with the corresponding control condition. Survival in each group was analyzed using a Kaplan-Meier method. A log-rank test was used for comparisons of survival data between groups. Statistical analysis was conducted using GraphPad Prism software (GraphPad 4.0) or IBM-SPSS version 19.0 software (IBM).

## SUPPLEMENTARY MATERIAL FIGURES AND TABLES


