# Supplementary material for: miR-192 suppresses leptomeningeal dissemination of medulloblastoma by modulating cell proliferation and anchoring through the regulation of DHFR, integrins, and CD47
Source: Oncotarget. 2015 Oct 25;6(41):43712–30. doi: 10.18632/oncotarget.6227 (PMC4791261; doi:10.18632/oncotarget.6227)
Supplement: Supplementary file 1 [file oncotarget-06-43712-s001.pdf]

miR-192 suppresses leptomeningeal dissemination of medulloblastoma by modulating cell proliferation and anchoring through the regulation of *DHFR*, integrins, and *CD47*

Supplementary Material

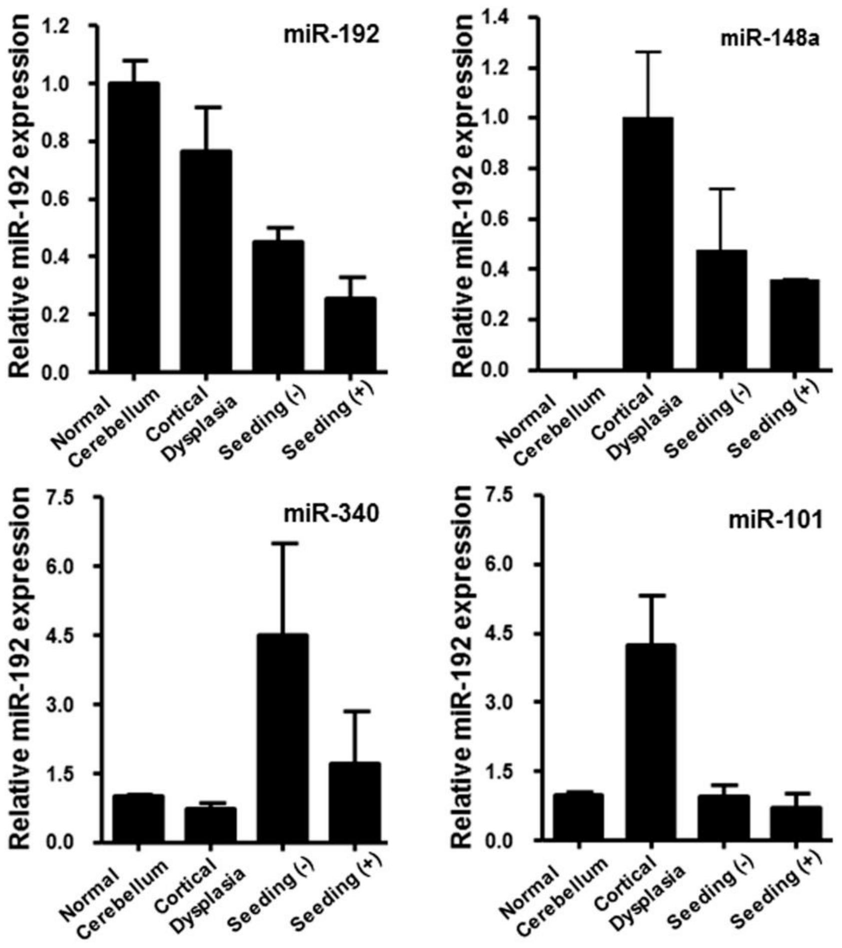

Supplementary Figure 1: Relative miRs expressions in medulloblastoma tissues, cell lines, normal cerebellum, and cortical dysplasia.

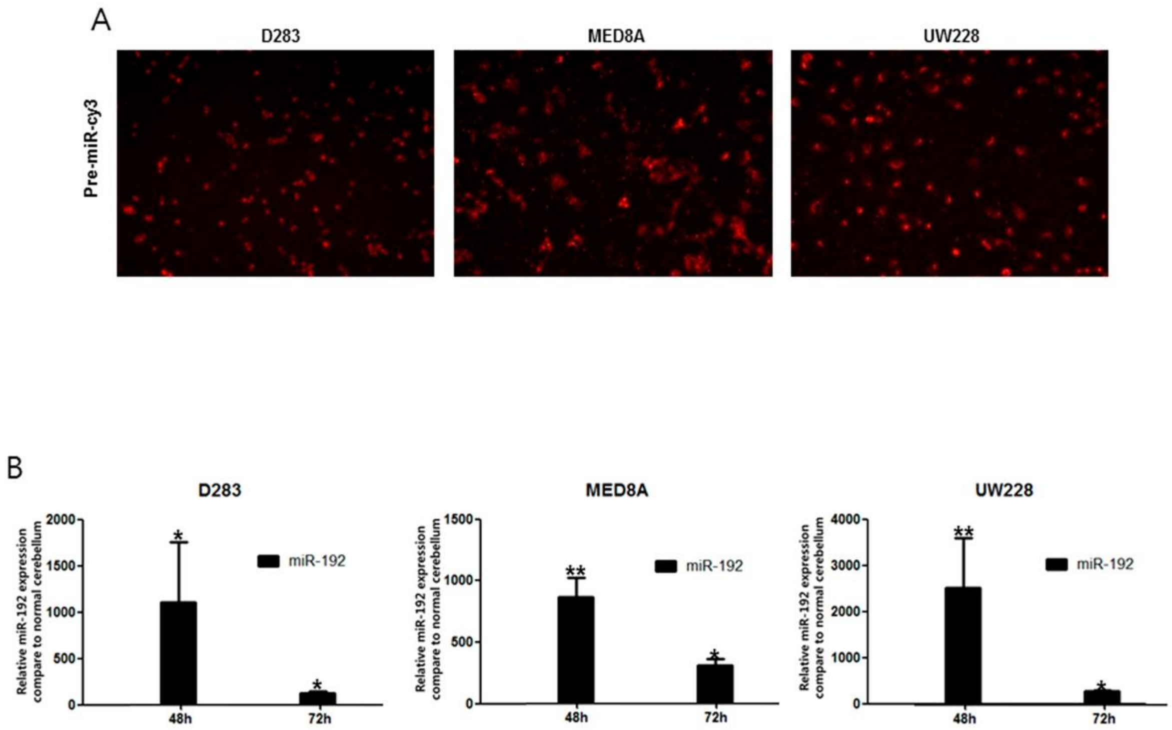

Supplementary Figure 2. Transfection of miR-192 in medulloblastoma cells. Transfection efficiency is monitored using Cy3-labeled pre-miRNA (A) and determined by Real-Time qRT-PCR (B).After transfection, miRNA-192 levels increased approximately 1100-fold in D283 ($P < 0.05$), 867-fold in MED8A ($P < 0.01$), and 2507-fold in UW228 ($P < 0.01$) at 48-hour and 127-fold in D283 ($P < 0.05$), 313-fold in MED8A ($P < 0.05$), and 267-fold in UW228 ($P < 0.05$) at 72-hour, compared to NC miRNA, respectively

Table S1. Differentially expressed miRs in the tumor seeding group compared to the tumor non-seeding group.

miR	Tumor seeding vs tumor non-seeding	
	$\log_2$ FC	P-value
hsa-miR-340-5p	− 0.595	0.008
hsa-miR-148a-3p	− 0.519	0.028
hsa-miR-101-3p	− 0.515	0.037
hsa-miR-192-5p	− 0.332	0.041
hsa-miR-34b-5p, or -3p	0.442	0.021
hsa-miR-32-3p	0.485	0.011
hsa-miR-483-3p	0.527	0.002
hsa-miR-574-3p	0.644	0.001
hsa-miR-574-5p	0.724	0.003
hsa-miR-630	0.725	0.031
hsa-miR-196a-5p	0.944	0.004
hsa-miR-494-3p, or -5p	1.512	0.042

FC: fold-change, hsa: Homo sapiens

Table S2. Characteristics of the patients with medulloblastoma (N=29)

Characteristic	CSF non-seeding group (N=20)	CSF seeding group (N=9)	P-value
Age at diagnosis (year), mean (range)	6.1 (1.1-13.0)	7.0 (0.7-15.0)	0.55
< 3	4 (20.0)	1 (11.1)	0.99
Female sex	8 (40.0)	4 (44.4)	0.99
Histopathological subtype			
Classic	12 (60.0)	8 (88.9)	0.26
Desmoplastic and nodular	3 (15.0)	0	
Anaplastic	5 (25.0)	1 (11.1)	
Molecular subtypes[a] according to Tayler *et al*.[2]			
WNT	0	0	
SHH	7	3	
Group 3	5	1	
Group 4	8	5	
Clinical follow-up period after surgery (months), mean	78.1 (17.0-167.0)	65.9 (11.0-150.0)	

(range)			
Final status at the last follow-up			
Overall survival (mean, 95% CI)	119.6 months (91.3-147.8)	70.5 months (31.9-109.1)	0.073
Progression-free survival (mean, 95% CI)	113.2 months (84.7-141.7)	69.4 months (30.8-108.1)	0.125

[a]The result is based on our previous study [49].
